# Complete Genome Sequence of Serratia marcescens Siphophage Serbin

**DOI:** 10.1128/MRA.00422-19

**Published:** 2019-05-09

**Authors:** Eric A. Williams, Helena Hopson, Andrea Rodriguez, Rohit Kongari, Rachele Bonasera, Adriana C. Hernandez-Morales, Mei Liu

**Affiliations:** a Center for Phage Technology, Texas A&M University, College Station, Texas, USA; DOE Joint Genome Institute

## Abstract

Serratia marcescens is an opportunistic human pathogen that is known to cause hospital-acquired respiratory and urinary tract infections. Here, we announce the complete genome sequence and the features of S. marcescens phage Serbin.

## ANNOUNCEMENT

Serratia marcescens is a Gram-negative rod-shaped bacterium present in abundance in the environment, and infections by this bacterium are often hospital acquired and localized to the respiratory, urinary, and gastrointestinal tracts (1–3). The study of S. marcescens phages may help control S. marcescens in hospital settings.

Phage Serbin was isolated using an S. marcescens strain from a pond water sample collected from College Station, Texas. Nutrient broth or agar (Difco) was used to culture the host bacteria and for phage enrichment at 37°C with aeration. Phage isolation and propagation were conducted by the soft agar overlay method (4). Phage genomic DNA was prepared using a modified Promega Wizard DNA cleanup kit protocol as described previously (5). Pooled indexed DNA libraries were prepared using the Illumina TruSeq Nano low-throughput (LT) kit, and a sequence was obtained with the Illumina MiSeq platform using the MiSeq v2 500-cycle reagent kit following the manufacturer’s instructions, producing 538,626 paired-end reads (250-bp read length) for the index containing the phage genome. The quality of the reads was checked in FastQC 0.11.5 (https://www.bioinformatics.babraham.ac.uk/projects/fastqc/), and reads were trimmed with FastX Toolkit 0.0.14 (http://hannonlab.cshl.edu/fastx_toolkit/download.html) and assembled in SPAdes 3.5.0 (6). The assembled genome was closed by PCR using primers (5′-CCCGACCGTTAAGACTGATTAC-3′ and 5′-CACCGAAGAGCACAAGAAGA-3′) facing away from the center of the assembled contig and by Sanger sequencing of the resulting product, with the contig sequence manually corrected to match the resulting Sanger sequencing read. Protein-coding genes were predicted using GLIMMER 3.0 (7) and MetaGeneAnnotator 1.0 (8) and corrected manually if needed. The tRNA genes were predicted using ARAGORN 2.36 (9). Protein functions were predicted by comparing predicted protein sequences to the NCBI nonredundant (nr) database using BLASTp 2.2.28 (10), and conserved domains were analyzed using InterProScan 5.15-54.0 (11). All analyses were performed under default settings using the CPT Galaxy (12) and Web Apollo (13) interfaces (https://cpt.tamu.edu).

Serbin has a 42,882-bp genome assembled with 1,968.4-fold coverage. There were 69 protein-coding genes identified, with only 25 having predictable functions. The genome has a GC content of 51.6% and a coding density of 96.6%. Using the progressiveMAUVE algorithm (version 2.4.0) (14), Serbin shows little recognizable DNA sequence similarity to any other phage in the NCBI nucleotide database. At the protein level, phage Serbin is related to a distinct Escherichia coli phage group reported previously, which includes the representative E. coli phage 9g (GenBank accession no. NC_024146) (15) and the more recently described four E. coli phages (16) JenP2 (accession no. KP719133), JenP1 (accession no. KP719132), JenK1 (accession no. KP719134), and CAjan (accession no. KP064094). As determined by a BLASTp search (expect [E] value of ≤10^−3^), Serbin shares 22 similar proteins with these groups of E. coli phages, but Serbin does not have the identifiable gene cluster encoding queuosine synthesis, which is a feature shared by the phages 9g, JenP2, JenP1, JenK1, and CAjan (15, 16). Three DNA biosynthesis genes, namely thymidylate kinase, thymidylate synthase, and cytidine deaminase, were found close to one another in a set. These three genes are involved with the metabolism of nucleotides, specifically that of thymidine and cytidine (17, 18). A lysis cassette was identified, with genes coding for a holin, endolysin (*N*-acetylmuramidase), and a partially embedded i-spanin/o-spanin motif.

### Data availability.

The genome sequence of phage Serbin was deposited under GenBank accession no. MK608336. The associated BioProject, SRA, and BioSample accession numbers are PRJNA222858, SRR8788533, and SAMN11260686, respectively.
